# DNA methylation regulates sclerostin (SOST) expression in osteoarthritic chondrocytes by bone morphogenetic protein 2 (BMP-2) induced changes in Smads binding affinity to the CpG region of *SOST* promoter

**DOI:** 10.1186/s13075-015-0674-6

**Published:** 2015-06-12

**Authors:** Ioanna Papathanasiou, Fotini Kostopoulou, Konstantinos N. Malizos, Aspasia Tsezou

**Affiliations:** Laboratory of Cytogenetics and Molecular Genetics, University of Thessaly, Faculty of Medicine, Biopolis, Larissa, 41500 Greece; Department of Orthopaedic Surgery, University of Thessaly, Faculty of Medicine, Biopolis, Larissa, 41500 Greece; Department of Biology, University of Thessaly, Faculty of Medicine, Biopolis, Larissa, 41500 Greece

## Abstract

**Introduction:**

Sclerostin (SOST), a soluble antagonist of Wnt signaling, is expressed in chondrocytes and contributes to chondrocytes’ hypertrophic differentiation; however its role in osteoarthritis (OA) pathogenesis is not well known. Based on our previous findings on the interaction between Wnt/β-catenin pathway and BMP-2 in OA, we aimed to investigate the role of DNA methylation and BMP-2 on SOST’s expression in OA chondrocytes.

**Methods:**

SOST mRNA and protein expression levels were investigated using real-time polymerase chain reaction (PCR) and Western blot, respectively. The methylation status of *SOST* promoter was analysed using methylation-specific PCR (MSP), quantitative methylation-specific PCR (qMSP) and bisulfite sequencing analysis. The effect of BMP-2 and 5’-Aza-2-deoxycytidine (5-AzadC) on SOST’s expression levels were investigated and Smad1/5/8 binding to *SOST* promoter was assessed by Chromatin Immunoprecipitation (ChΙP).

**Results:**

We observed that SOST’s expression was upregulated in OA chondrocytes compared to normal. Moreover, we found that the CpG region of *SOST* promoter was hypomethylated in OA chondrocytes and 5-AzadC treatment in normal chondrocytes resulted in decreased *SOST* methylation, whereas its expression was upregulated. BMP-2 treatment in 5-AzadC-treated normal chondrocytes resulted in SOST upregulation, which was mediated through Smad 1/5/8 binding on the CpG region of *SOST* promoter.

**Conclusions:**

We report novel findings that DNA methylation regulates SOST’s expression in OA, by changing Smad 1/5/8 binding affinity to *SOST* promoter, providing evidence that changes in DNA methylation pattern could underlie changes in genes’ expression observed in OA.

## Introduction

Osteoarthritis (OA), a chronic degenerative disease of the joints, is a major health burden linked to high morbidity in the aging population [1, 2]. The central pathological features of OA are the progressive degradation of articular cartilage, new bone formation at joint margins (osteophytes) and changes in subchondral bone structure (sclerosis) [3]. OA is considered a multifactorial disease and several risk factors contribute to its pathogenesis, including genetic predisposition, aging, obesity and joint malignment [2, 4].

Articular chondrocytes may be the most important cells that are involved in OA pathogenesis [5, 6]. The disruption of matrix equilibrium between synthesis and degradation of extracellular matrix (ECM) components and progressive loss of cartilage tissue are associated with changes in their anabolic and catabolic activities following exposure to multiple signals [7, 8]. Recently, it was demonstrated that one of the genes that are deregulated in OA chondrocytes is *SOST* [9]. Sclerostin (SOST), encoded by the *SOST* gene, is specifically expressed by osteocytes and is involved in bone homeostasis [10, 11]. SOST is a soluble antagonist of Wnt signaling [12] and it has been demonstrated that *SOST* loss-of-function mutations cause abnormal skeletal phenotypes in humans, characterized by high bone mineral density [13, 14], whereas transgenic mice that overexpress SOST are osteopenic due to reduced bone formation [15]. In OA, which is characterized by new bone formation, it has been reported that SOST is implicated in OA disease processes in both bone and cartilage with opposing effects, by promoting subchondral bone sclerosis while inhibiting cartilage degradation [9].

Besides the well-known role of SOST as a Wnt signaling inhibitor, it has been recently suggested that SOST interacts with other signaling pathways, such as bone morphogenic proteins (BMPs) and affects the biology of the skeleton [16–18]. The canonical BMP-Smad pathway induces human mesenchymal stem cells to differentiate into chondrocytes and osteoblasts and BMP-2 is a crucial local factor responsible for chondrocyte proliferation and maturation during endochondral ossification [19, 20]. Although the interaction between SOST and BMPs is not yet clear, it has been shown that in osteoblasts, SOST binds to BMPs and modulates the activity of osteoblastic cells by reducing the expression of alkaline phosphatase (ALP), synthesis of type I collagen, and mineralization [15]. Despite the role of SOST as a Wnt and BMP signaling inhibitor, little is known about its gene regulation. Previous studies have reported that different molecular mechanisms are able to modulate SOST expression, among which BMPs and parathyroid hormone (PTH) [21–24]. Moreover, recent studies point towards the involvement of DNA methylation in the regulation of SOST expression in human osteocytes and bone cells [18, 25, 26]. In the present study, we sought to investigate first whether DNA methylation regulates SOST expression in OA chondrocytes, and the role of BMP-2 on changes in SOST expression in OA.

## Materials and methods

### Bioinformatic analysis

The 1,500 bp upstream of the *SOST* transcript start site (TSS) were obtained from Ensembl genome browser and putative CpG islands were identified using Metlyl Primer Express software v1.0 (available from Applied Biosystems). A CpG island was defined as a region of at least 200 bp, with GC content greater than 50 %, and observed-to-expected (O/E) CpG ratio >0.6 [27]. CpG islands were tested for Smad binding sites (SBEs, 5’-GCCGnGCG-3’) using ChIP bioinformatics tools.

### Patients and cartilage samples

Articular cartilage samples were obtained from femoral condyles and tibial plateaus of 14 patients (11 female/3 male; mean age 67.8 ± 9.6 years) with primary hypertrophic OA, undergoing knee replacement surgery at the Orthopaedics Department of the University Hospital of Larissa. Radiographs were obtained before surgery and graded using the Kellgren-Lawrence system according to the following criteria: grade 1 (doubtful narrowing of joint space and possible osteophytes), grade 2 (definite osteophytes and possible narrowing of joint space), grade 3 (moderate multiple osteophytes, definite narrowing of joint space and some sclerosis and possible deformity of bone ends) and grade 4 (large osteophytes, marked narrowing of joint space, severe sclerosis and definite deformity of bone ends). All patients had a Kellgren-Lawrence grade ≥3. The assessment of the radiographs by two independent expert observers was blinded. Normal articular cartilage was obtained from 10 individuals (7 female/3 male; mean age 56.9 ± 10.8 years), undergoing knee fracture repair surgery, with no history of joint disease and who did not show clinical manifestations compatible with OA when specifically explored by radiography. Written informed consent was obtained from all individuals in the study. The study protocol conformed to the ethical guidelines of the 1975 Declaration of Helsinki as reflected in a priori approval by the local ethical committee of the University Hospital of Larissa.

### Primary cultures of normal and OA human articular chondrocytes

Articular cartilage was dissected and subjected to sequential digestion with 1 mg/ml pronase and 1 mg/ml collagenase P (Roche Applied Science, Mannheim, Germany). Chondrocytes were counted and checked for viability using trypan blue staining. More than 95 % of the cells were viable after isolation. Isolated chondrocytes from individual specimens were separately cultured with DMEM/F-12 (GIBCO, Life Technologies, Paisley, UK) plus 5 % FBS (Invitrogen, Life Technologies, Paisley, UK) at 37 °C under a humidified 5 % CO_2_ atmosphere until reaching confluence for 4–6 days. Half of cultured chondrocytes were then harvested by trypinization and were used for DNA, RNA and protein extraction. The other half was cultured again without treatment until confluence for 1 week (passage-1 chondrocytes).

Passage-1 normal and OA chondrocytes were seeded on six-well plates at 3 × 10^5^ cells/well and 3 days post-seeding cells were treated with 50 ng/ml of BMP-2 (Sigma-Aldrich, MO, USA) for 24 and 48 h or with 5 μM 5-AzadC (Sigma-Aldrich) in dimethyl sulfoxide (DMSO). Media containing DMSO or DMSO+5-AzadC was exchanged daily and lasted for 5 days. Moreover, for BMP-2 experiments, chondrocytes were treated with or without 5-AzadC for 3 days, then media was removed and 50 ng/ml of BMP-2 was added for 48 h.

### RNA extraction and quantification of mRNA expression

Total cellular RNA was extracted from cultured chondrocytes using Trizol reagent (Invitrogen, Life Technologies, Paisley, UK). Preservation of 28S and 18S ribosomal RNA (rRNA) species was used to assess RNA integrity. All the samples included the study were with prominent 28S and 18S rRNA components. The yield was quantified spectrophotometrically. Transcription of 1 μg RNA to cDNA was performed using SuperScript III reverse transcriptase (Invitrogen, Life Technologies, Paisley, UK) and random primers (Invitrogen, Life Technologies, Paisley, UK). Quantification of SOST mRNA expression was performed by real-time PCR (ABI 7300, Applied Biosystems, Foster, CA, USA). The oligonucleotide primers used for SOST amplification are shown in Table 1. Reactions were done in triplicate using 2 μl of cDNA per reaction. Real-time PCR validation was carried out using the 2^-ΔΔCT^ method. Normalized gene expression values for each gene based on cycle threshold (C_T_) values for each of the genes and the housekeeping gene glyceraldehyde 3-phosphate dehydrogenase (*GAPDH*) were generated.

**Table 1:** Primer sequences for PCR, real-time PCR, methylation-specific PCR (MSP), quantitative MSP (Qmsp) and bisulfite (Bis.) sequencing analysis

Name	Sequence (5′-3)	Experiment
M-SOST-forward	GAATAGGTCGGGTTTAGTTTC	MSP, qMSP
M-SOST-reverse	ACCTCCCACGTACTAACGA	MSP, qMSP
U-SOST-forward	GGAATAGGTTGGGTTTAGTTTT	MSP, qMSP
U-SOST-reverse	CACCTCCCACATACTAACAA	MSP, qMSP
SOST-forward	CCGGAGCTGGAGAACAACAAG	RT-PCR
SOST-reverse	GGTGTGCTCCGGCCAGTGC	RT-PCR
Promoter SOST-forward	GGGACCAATGGGATTTCTTT	PCR
Promoter SOST-reverse	TGAGCTCCGGCTTTTAATTG	PCR
BSP SOST-forward	TTATTTGTTGGTGGGGTGATAA	Bis. sequencing
BSP SOST-reverse	ACAAAACCCAAACCTACTCTCC	Bis. sequencing

### Protein extraction and western blot analysis

Chondrocytes were lysed using radioimmunoprecipitation assay (RIPA) buffer containing 10 mM Tris (pH 7.5), 150 mM NaCl, 1 % Triton X-100, 1 % sodium deoxycholate, 0.1 % SDS, 1 mM EDTA, and a cocktail of protease inhibitors. Protein concentration was quantified using the Bio-Rad Bradford protein assay (Bio-Rad Protein Assay, BioRad, Hercules, CA, USA) with bovine serum albumen as standard. Cell lysates from chondrocytes were electrophoresed and separated on 12 % acrylamide gels and transferred to PVDF membranes (Millipore, Billerica, MA, USA). The membrane was probed with anti- SOST (1:100 dilution) (Novus biologicals, CO, USA) and signal was detected using anti-rabbit immunoglobulin IgG conjugated with horseradish peroxidase (1:10.000 dilution) (Invitrogen, Life Technologies, Paisley, UK). The results were normalized using anti-β-actin polyclonal antibody (1:3.000 dilution) (Sigma-Aldrich, MO, USA). PVDF membranes were then exposed to photographic film and western blot bands from several different blots were quantified using the NIH Scion Image according to the software guidelines.

### DNA methylation analysis by MSP

Genomic DNA was extracted from normal and OA cultured chondrocytes using the Genomic DNA Isolation Kit (Qiagen, Valencia, CA, USA) and was treated with bisulfite conversion reagents using the MethylCode™ Bisulfite Conversion Kit (Invitrogen, Life Technologies, Paisley, UK) according to the manufacturer’s instruction. The region of interest in the *SOST* promoter was amplified by PCR using primers for MS-PCR derived from the Methlyl Primer Express (software v1.0) (Table 1). PCR reaction was confirmed by electrophoresis in a 3 % agarose gel and was stained with ethidium bromide. Quantification analysis of bands was performed using the NIH Scion Image according to the software guidelines.

### DNA methylation analysis by qMSP

Quantitative methylation-specific PCR (qMSP) for the CpG island of the *SOST* promoter was performed using a real-time PCR instrument (ABI 7300, Applied Biosystems, Foster, CA, USA). In the qMSP reaction, 2 μl of bisulfite-treated genomic DNA were amplified with 2 × EpiTect Master Mix (Qiagen, Valencia, CA, USA) and 0,75 μΜ primers (Table 1) in a total volume of 25 μl. Amplification conditions were: 95 °C for 5 minutes, followed by 40 cycles of 95 °C for 10 s, 55 °C for 30 s, and 72 °C for 27 s, with a final extension of 72 °C for 10 minutes. DNA methylation values were calculated by interpolating the cycle threshold gap (CtU-CtM) in a standard curve, conducted using mixtures of methylated and unmethylated human control samples with 0 %, 10 %, 25 %, 50 %, 75 %, 90 % and 100 % methylated DNA (Qiagen, Valencia, CA, USA).

### Bisulfite DNA sequencing analysis

Bisulfite-treated DNA was amplified by PCR using primers for BSP-PCR derived from the Methlyl Primer Express (software v1.0) (Table 1).). In the PCR reaction, 2 μl of bisulfite-treated genomic DNA were amplified with 10 × PCR buffer, 400 μΜ dNTPs, 1 U of AmpliTaq Gold DNA polymerase (Applied Biosystems, Foster, CA, USA) and 0,5 μΜ of each primer in a total volume of 25 μl. Amplification conditions were: 95 °C for 10 minutes, followed by 40 cycles of 95 °C for 10 s, 54 °C for 30 s, and 72 °C for 1 minute, with a final extension of 72 °C for 5 minutes. PCR products were cleaned using QIAquick PCR Purification kit (Qiagen, Valencia, CA, USA) and then were sequenced using a Bigdye terminator v3.1 cycle sequencing kit (Applied Biosystems, Foster, CA, USA) and analyzed on the ABI 3130 Genetic Analyzer (Applied Biosystems). Sequencing was performed using the forward primer and the methylation percentage for each CpG site in the CpG island was quantified by measuring the ratio between peak height values of cytosine (C) and thymine (T), yielding the basic equation for the methylation percentage to be (C/(C + T) *100) [28].

### Chromatin immunoprecipitation (ChIP) assay

ChIP was performed using a ChIP assay kit (Upstate USA, Inc., Charlottesville, VA, USA) on normal and OA chondrocytes. Cell lysates were pre-cleared by incubation with G-Sepharose beads and were incubated with monoclonal antibody Smad-1/5/8 (Cell signalling Technology, Boston, MA, USA) overnight at 4 °C. Antibody human purified IgG was used as control (R&D Systems, McKinley Place, MN, USA). The immunoprecipitated DNAs were used for PCR amplification. The primers were designed according to the nucleotide sequence of *SOST* promoter and the PCR fragment covered 250–400 bp of the promoter. Table 1 shows the primer sets that amplify the promoter region containing putative sites as observed after bioinformatic analysis. The PCR products were fractionated on 3 % agarose gels and were stained with ethidium bromide. Quantification analysis of bands was performed using the NIH Scion Image according to the software guidelines.

### Statistical analysis

Data were analyzed using the SPPS software 20. Statistical significance was determined using Student’s *t* test and a confidence level of 95 % (*p* <0.05).

## Results

### SOST mRNA and protein expression levels are increased in OA chondrocytes

**Fig. 1:**
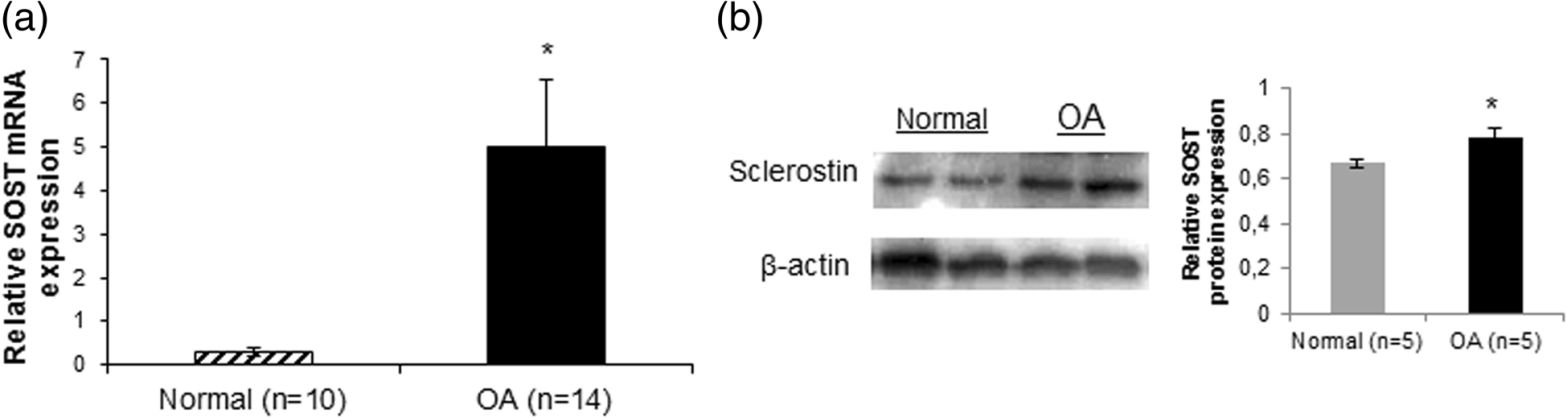
SOST mRNA and protein expression levels in normal and osteoarthritis (*OA*) chondrocytes. **a** Quantitative SOST mRNA expression in cultured normal (n = 10) and OA chondrocytes (n = 14). GAPDH was used for normalization of the real-time PCR data (*error bars* = standard error, *p = 0.005). **b** Representative western blot of SOST protein expression in cultured normal and OA chondrocytes and a bar graph showing relative SOST protein expression normalized to β-actin in normal (n = 5) and OA chondrocytes (n = 5) (*error bars* = standard error, *p = 0.038)

Using real-time PCR, we found higher SOST mRNA expression levels in OA compared to normal chondrocytes (*p* = 0.005) (Fig. 1a). SOST protein levels confirmed our real-time PCR findings. Western blot analysis revealed that SOST protein expression is elevated in OA chondrocytes compared to normal (*p* = 0.038) (Fig. 1b).

### DNA methylation status of *SOST* promoter is different between normal and OA chondrocytes

**Fig. 2:**
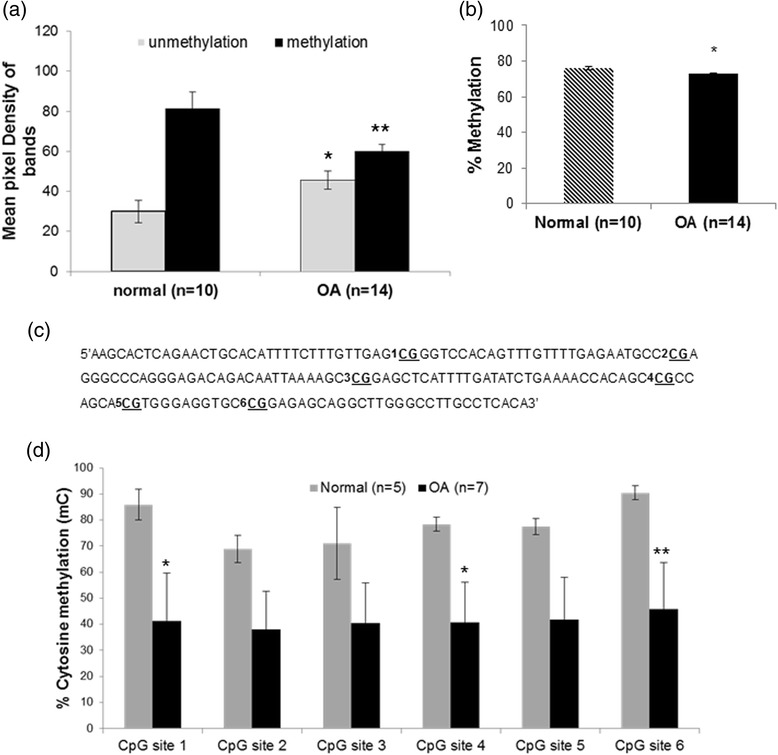
DNA methylation status of the *SOST* promoter in normal and osteoarthritis (*OA*) chondrocytes. **a** DNA methylation status of CpG island located upstream of the transcript start site (*TSS*) in the region of the *SOST* promoter in cultured normal (n = 10) and OA chondrocytes (n = 14) (*error bars* = standard error, *p = 0.05 versus normal unmethylation status, **p = 0.043 versus normal methylation status). **b** DNA methylation values in the CpG-rich region of the *SOST* promoter in cultured normal (n = 10) and OA chondrocytes (n = 10) by qMSP (error bars = standard error, *p = 0.004). **c** Schematic representation of the CpG-rich region of the *SOST* promoter showing the six CpG sites that were analyzed using the bisulfite DNA sequencing method. **d** The percentage of cytosine methylation of each CpG site located at the CpG region of the *SOST* promoter in cultured normal (n = 5) and OA chondrocytes (n = 7), after bisulfite DNA sequencing analysis (error bars = standard error, *p = 0.05 and **p = 0.046)

Taking into consideration recent reports that demonstrated involvement of epigenetics in the regulation of SOSTs expression, we tested whether the DNA methylation status of the SOST promoter is different between normal and OA chondrocytes. Bioinformatic analysis revealed the presence of two CpG islands surrounding the TSS of the *SOST* gene. One CpG island was located upstream of the TSS and the other within exon 1, downstream of the transcription start codon. We then tested the CpG islands for the presence of transcription factors binding sites and especially Smad binding sites, as we wanted to investigate the role of BMP-2, mediated through Smad proteins, on SOST expression. As we found Smad binding sites only in the first CpG island located upstream of the TSS in the region of the *SOST* promoter, we investigated the methylation status of this CpG island. This CpG island was identified between −516 bp and −256 bp upstream of the TSS, as a region of DNA spanning over 200 bp with a GC content over 50 %. Using MSP technology, we evaluated the methylation status of this region in genomic DNA isolated from normal and OA chondrocytes. Our results showed a significant difference in the methylation status between normal and OA chondrocytes in this CpG island located at the *SOST* promoter (Fig. 2a). Moreover, analysis by qMSP demonstrated that this CpG-rich region at the *SOST* promoter was highly methylated in normal chondrocytes (76,18 % ± 0,842) compared to OA chondrocytes (72,68 % ± 0,654) (*p* = 0.004) (Fig. 2b). To identify the particular CpG sites in this region whose methylation status is associated with SOST expression, we analyzed six CpG dinucleotides in the CpG island located at the *SOST* promoter using bisulfite DNA sequencing. We found that CpG sites 1, 4 and 6 were highly methylated (*p* = 0.05, *p* = 0.05 and *p* = 0.046, respectively) in normal compared to OA chondrocytes (Fig. 2c and d).

### SOST expression is induced by 5-AzadC through modification of DNA methylation status

**Fig. 3:**
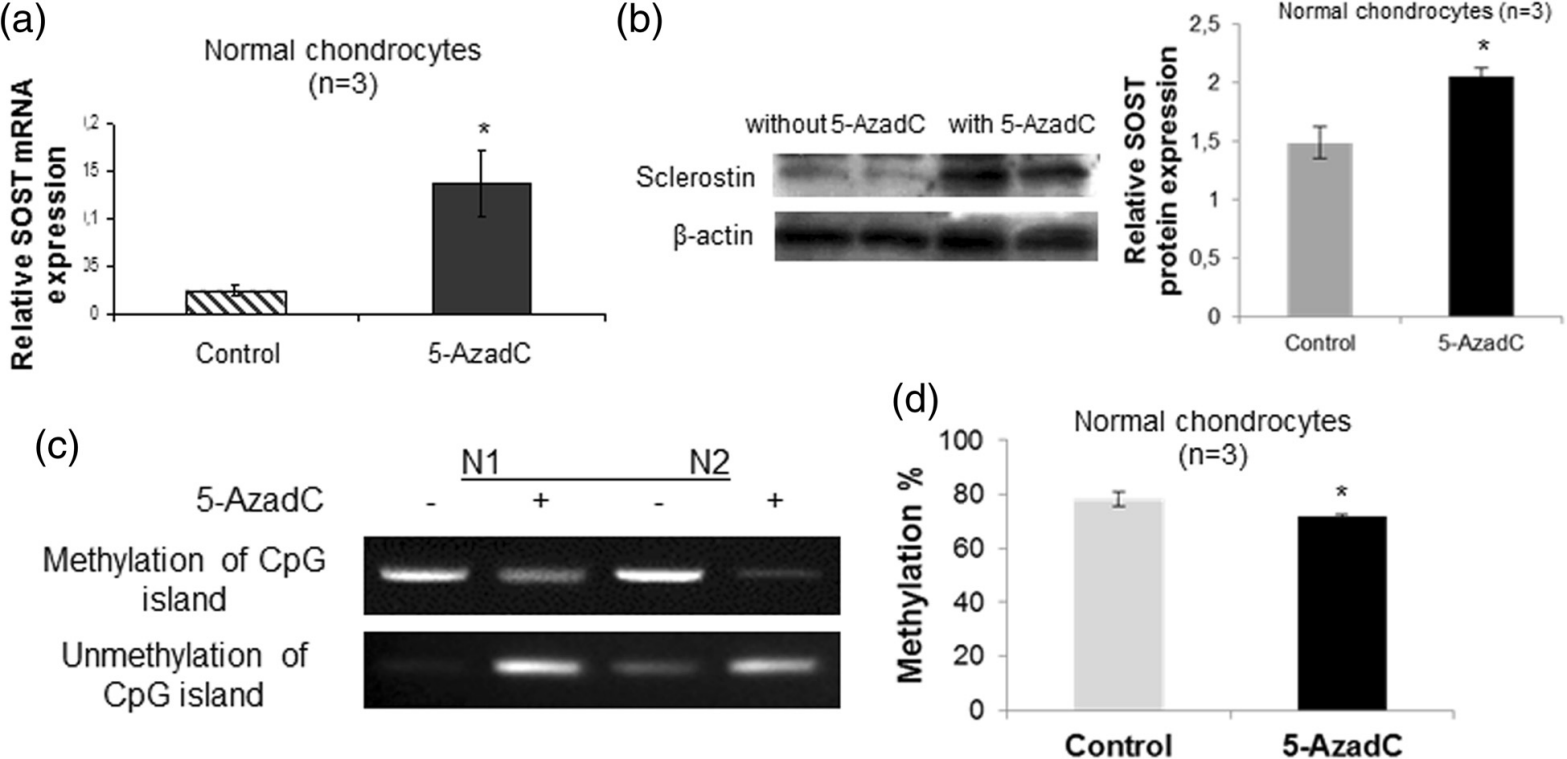
Effect of 5-AzadC treatment on SOST expression and DNA methylation status in the CpG-rich region of the *SOST* promoter. **a** Quantitative SOST mRNA expression in cultured normal chondrocytes (n = 3) after treatment with 5 μM 5-AzadC. GAPDH was used for normalization of the real-time PCR data (*error bars* = standard error, *p = 0.041). **b** Representative western blot of SOST protein levels in cultured normal chondrocytes after treatment with 5 μM 5-AzadC and a bar graph showing relative SOST protein expression normalized to β-actin in 5-AzadC-treated normal chondrocytes (n = 3) (*error bars* = standard error, *p = 0.009). **c** DNA methylation and unmethylation status of the *SOST* promoter in cultured normal chondrocytes after treatment with 5 μM 5-AzadC. **d** DNA methylation values in CpG-rich region of the *SOST* promoter in cultured normal chondrocytes (n = 3) after treatment with 5 μM 5-AzadC by quantitative methylation-specific PCR (*error bars* = standard error, *p = 0.032)

To confirm the causal inverse association between *SOST* promoter methylation and gene expression, we evaluated the ability of the demethylating agent 5-AzadC to promote SOST expression in chondrocytes. Normal chondrocytes, for which the promoter was found to be methylated, were treated with 5-AzadC and subsequently we evaluated SOST mRNA and protein levels by real-time PCR and western blot analysis, respectively. We found that SOST mRNA and protein levels (*p* = 0.041, *p* = 0.009, respectively) were markedly increased in 5-AzadC-treated cells compared to untreated (Fig. 3a, b) and this 5-AzadC-induced change in gene expression was associated with a decrease in DNA methylation in the CpG-rich region of the *SOST* promoter (*p* = 0.032) (Fig. 3c, d).

### DNA methylation contributes to regulation of SOST expression by impairing the binding affinity of Smad 1/5/8 transcription factors in its promoter

**Fig. 4:**
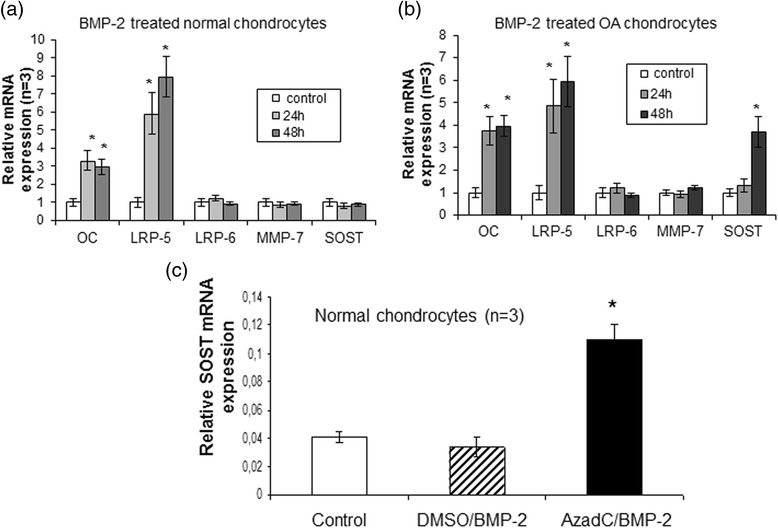
Effect of bone morphogenic protein 2 (*BMP*-*2*) and 5-AzadC treatment on SOST expression. **a** and **b** Efects of BMP-2 on SOST mRNA expression levels in cultured normal (n = 3) and osteoarthritis (*OA*) chondrocytes (n = 3). Osteocalcin and LRP-5 used as positive controls that upregulated by BMP-2 and LRP-6 and MMP-7 used as negative controls that not regulated by BMP-2(*error bars* = standard error, *p = 0.004). **c** Detection of SOST mRNA expression levels by real time PCR after BMP-2 treatment in cultured normal chondrocytes (n = 3) with or without 5-AzadC. GAPDH was used for normalization of the real-time PCR data (*error bars* = standard error, *p = 0.001 versus control and DMSO/BMP-2 treatment). *MMP* matrix metalloproteinase, *LRP* low-density lipoprotein receptor-related protein

**Fig. 5:**
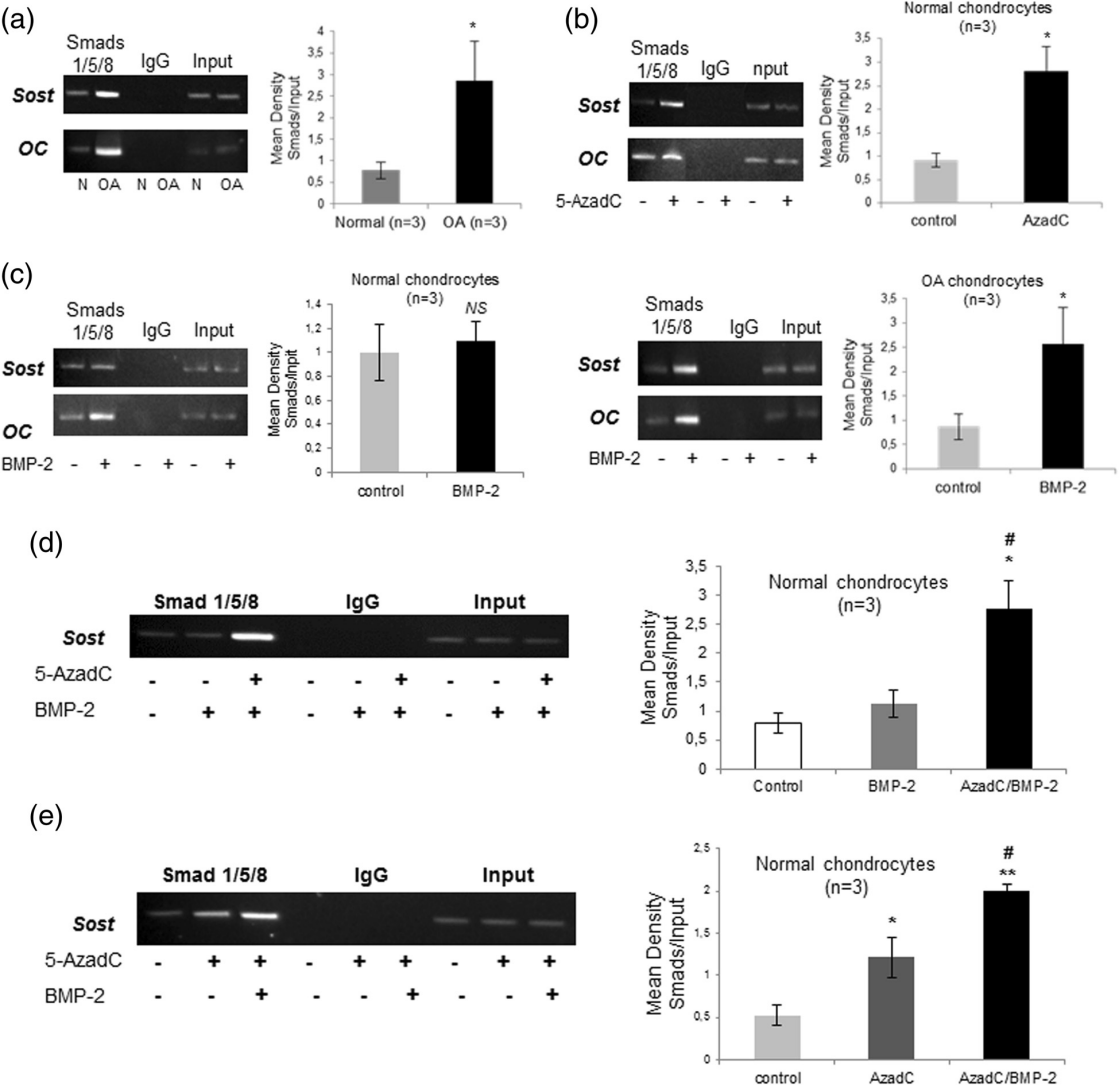
Occupancy of the *SOST* promoter by Smad1/5/8 by chromatin immunoprecipitation (ChIP) analysis. **a** Representative gel of Smad1/5/8 binding on the *SOST* promoter in cultured normal and osteoarthritis (*OA*) chondrocytes and densitometric analysis of the band intensity in cultured normal (n = 3) and OA chondrocytes (n = 3) (*error bars* = standard error, *p = 0.05 versus normal chondrocytes). **b** Representative gel of Smad1/5/8 binding on the *SOST* promoter in 5-AzadC- treated and untreated normal chondrocytes and densitometric analysis of the band intensity in three different samples (n = 3) (*error bars* = standard error, *p = 0.05 versus control) **c** Representative gel of Smad1/5/8 binding on the *SOST* promoter in bone morphogenic protein 2 (*BMP*-*2*)-treated and untreated normal and OA chondrocytes and densitometric analysis of the band intensity in normal (n = 3) and OA samples (n = 3) (error bars = standard error, *p = 0.05 versus control, *NS* = not significant). **d** Representative gel of Smad1/5/8 binding on the *SOST* promoter after BMP-2 treatment in cultured normal chondrocytes with or without 5-AzadC and densitometric analysis of the band intensity in three different samples (n = 3). (*error bars* = standard error, *p = 0.05 versus control and #*p* = 0.05 versus BMP-2 treatment). Input chromatin used as positive control and IgG as negative control. **e** Representative gel of Smad1/5/8 binding on the *SOST* promoter after 5-AzadC treatment in cultured normal chondrocytes with or without BMP-2 and densitometric analysis of the band intensity in three different samples (n = 3) (*error bars* = standard error, *p = 0.05 versus control, #*p* = 0.05 versus control and **p = 0.05 versus 5-AzadC treatment)

BMP-2 has been reported to play a significant role in the regulation of SOST expression in bone. To gain insight into the molecular mechanisms underlying SOST expression in OA chondrocytes, we examined the effect of BMP-2 on SOST expression in normal and OA chondrocytes. We found that BMP-2 treatment resulted in significant induction of SOST expression in OA chondrocytes (*p* = 0.004), but not in normal chondrocytes (Fig. 4a and b). However, SOST expression levels were upregulated after BMP-2 treatment in 5-AzadC-treated normal chondrocytes (Fig. 4c) (*p* = 0.001). To further investigate the intracellular signaling pathway involved in BMP-2-induced SOST expression, normal, OA, BMP-2 and/or 5-AzadC-treated chondrocytes were subjected to ChIP assay using an antibody against Smad-1/5/8 and we tested whether Smads bind to the *SOST* promoter via Smad binding elements. We found that the *SOST* promoter contains a conserved Smad binding site in the CpG island located upstream of the TSS and that Smad1/5/8 binding was enhanced in OA compared to normal chondrocytes (*p* = 0.05) and in BMP-2-treated OA compared to untreated chondrocytes (*p* = 0.05) (Fig. 5a and c). Moreover, stronger binding of Smad1/5/8 was observed in 5-AzadC-treated normal chondrocytes compared to untreated (Fig. 5b) (*p* = 0.05) and this affinity was significantly increased in BMP-2/5-AzadC-treated normal chondrocytes compared to 5-AzadC-treated, BMP-2-treated and untreated chondrocytes (Fig. 5d and e) (*p* = 0.05). No difference was observed between BMP-2-treated and untreated normal chondrocytes (Fig. 5c).

## Discussion

SOST is a potent Wnt antagonist and a key regulator of bone metabolism [9, 29]. OA is characterized by changes in bone matrix composition and metabolism, however, the role of SOST in OA pathogenesis is not well known. In the present study, we found that SOST is upregulated in OA chondrocytes compared to normal, in agreement with recent studies demonstrating that SOST is expressed by articular chondrocytes and not only by bone cells in end-stage OA [9, 30, 31]. However, Roudier et al. showed that there is no difference in SOST expression between normal and OA cartilage but a strong SOST staining only in chondrocyte clusters that are often observed in damaged OA articular cartilage [32]. Our results showed an association between SOST expression and OA, however further investigation is necessary to determine whether upregulation of SOST expression in OA chondrocytes is a causal factor in OA pathogenesis or a result of the OA process.

It is known that DNA methylation patterns change with increasing age and age-dependent hypomethylation may contribute to pathological processes such as OA, an age-related disease [33]. In an attempt to investigate the role of DNA methylation on SOST expression in OA, we investigated the methylation status of the *SOST* gene and observed, for the first time, that the *SOST* promoter was hypermethylated in normal chondrocytes and hypomethylated in OA chondrocytes, suggesting the involvement of epigenetic mechanisms in the regulation of SOST expression in OA. However, additional functional studies are needed to clarify whether these DNA methylation changes in the *SOST* promoter are biologically relevant. In general, changes in DNA methylation have been shown to have an impact on OA pathology, as several studies have demonstrated a different methylation profile between OA and normal cartilage [34–36].

Further evidence for a relationship between DNA methylation and gene expression was obtained after treatment of chondrocytes with 5-AzadC, a potent inhibitor of DNA methylation. We found that 5-AzadC treatment in normal chondrocytes resulted in upregulation of SOST’s expression through altered methylation status of the *SOST* promoter, suggesting that this region of the promoter influences SOST expression in chondrocytes. Previous studies have demonstrated that DNA methylation contributes to the regulation of SOST’s expression in human osteocytes and bone cells [18, 25, 26, 37], suggesting a common molecular mechanism of SOST gene expression in different cell types.

Besides DNA methylation, other factors, such as growth factors and hormones can modulate SOST expression. BMP-2 is a growth factor which plays an important role in cartilage and bone homeostasis and it has been demonstrated that BMP-2 contributes to the regulation of SOST expression [16–18]. We found that treatment with BMP-2 resulted in significant increase in SOST expression in OA chondrocytes but not in normal. However, SOST expression was upregulated after BMP-2 treatment in 5-AzadC-treated normal chondrocytes, suggesting that DNA methylation may impair the binding of transcriptional factors, especially Smad binding in the *SOST* promoter. By using the ChIP assay, we demonstrated the existence of a Smad binding site in the CpG region located upstream of the TSS and found that the binding affinity was decreased in the methylated promoter, as the CpG dinucleotide, which were shown to be methylated, were located at/or near the Smad binding site. In addition, we observed that BMP-2 induced SOST expression in 5-AzadC-treated normal chondrocytes, which correlated with stronger binding affinity of Smads in the *SOST* promoter, suggesting that ΒΜΡ-2 and the methylation status of the *SOST* promoter regulate SOST transcriptional levels. In a recent study, it was demonstrated that BMPs stimulate SOST expression in human bone cells by a mechanism involving BMPR1A receptor and the downstream Smad-dependent pathway, suggesting a direct influence of BMPs on SOST transcriptional levels [18]. Moreover, Thillainadesan et al. showed an association between DNA methylation status and Smad binding, as they demonstrated that transforming growth factor (TGF)-β signaling activation resulted in DNA demethylation of p15^ink4b^ gene and subsequently increased recruitment of SMAD2/3 on p15^ink4b^ gene promoter [38].

SOST has become an attractive target for the treatment of osteoporosis and other skeletal diseases associated with low bone mineral density and increased fracture risk [31, 39]. Preclinical studies have demonstrated that antisclerostin therapy results in increased bone formation and bone mass in animal models [40–42]. Moreover, recent human clinical trials with sclerostin-neutralizing monoclonal antibody (Scl-Ab) therapy have shown beneficial effects on bone formation and resorption markers in healthy men and postmenopausal women [43]. However, in OA the findings in preclinical studies using antisclerostin therapy have been disappointing, as genetic absence of sclerostin or antisclerostin therapy with monoclonal antibody had no impact on articular cartilage remodeling in animals with age-dependent OA or post-traumatic OA, respectively [32]. On the other hand, a recent study showed that plasma and synovial fluid SOST levels are inversely associated with radiographic severity in knee OA, suggesting SOST as a protective factor in OA and a possible biochemical marker of knee OA for reflecting the degenerative process of primary knee OA [44]. Based on the above reports and our results it can be suggested that SOST may play a role in OA pathogenesis but its impact on cartilage biology and extracellular matrix degradation may be less powerful compared to its major regulatory role in bone mass.

## Conclusion

Our novel data strongly suggest that BMP-2 signaling modulates *SOST* transcription in OA through changes in Smad 1/5/8 binding affinity to the CpG region located upstream of the TSS in the *SOST* gene, pointing towards the involvement of DNA methylation in SOST expression in OA.

## Abbreviations

5-AzadC: 5’-Aza-2-deoxycytidine
BMP-2: bone morphogenetic protein 2
bp: base pairs
ChΙP: chromatin immunoprecipitation
DMEM/F-12: Dulbecco’s Modified Eagles Medium/Ham’s F-12
FBS: fetal bovine serum
GAPDH: glyceraldehyde 3-phosphate dehydrogenase
MSP: methylation-specific polymerase chain reaction
OA: osteoarthritis
PCR: polymerase chain reaction
qMSP: quantitative methylation-specific polymerase chain reaction
SBE: Smad binding element
SOST: sclerostin
TSS: transcript start site
